# Visceral leishmaniasis triggering an adult‐onset Still's disease: a unique case

**DOI:** 10.1002/ccr3.1266

**Published:** 2017-11-13

**Authors:** Silvia Spoto, Sebastiano Costantino, Emanuele Valeriani, Marta Fogolari, Eleonora Cella, Giordano Dicuonzo, Massimo Ciccozzi, Silvia Angeletti

**Affiliations:** ^1^ Internal Medicine Department University Hospital Campus Bio‐Medico Rome Italy; ^2^ Unit of Clinical Laboratory Science University Campus Bio‐Medico of Rome Rome Italy; ^3^ Department of Public Health and Infectious Diseases “Sapienza”, Policlinico Umberto I University of Rome Rome Italy

**Keywords:** Adult‐onset Still's disease, fever of unknown origin, visceral leishmaniasis

## Abstract

Adult‐onset Still's disease (AOSD) due to visceral leishmaniasis (VL) has not been previously reported. This case report analyzes a single episode of AOSD probably due to a visceral leishmaniasis successfully treated with pentamidine isethionate and prednisone.

## Introduction

Fever of unknown origin (FUO) is a persistent febrile illness without an established etiology despite intensive diagnostic evaluation. Visceral leishmaniasis (VL) and adult‐onset Still's disease (AOSD) are both causes of FUO. We describe a case of AOSD probably due to a visceral leishmaniasis.

## Case Report

A 30‐year‐old woman was admitted to our Internal Medicine Department because of a continuous remittent fever (up to 40°C) present for a month, profuse sweating, pelvic girdle arthralgia and myalgia, and sore throat with dry cough. The patient always lived in Trapani and did not travel recently. She had been in her usual health until a month before admission in Trapani Hospital, where laboratory tests showed neutrophilic leukocytosis, normochromic normocytic anemia, erythrocyte sedimentation rate (ESR) of 120 mm/h, C‐reactive protein (CRP) of 193 mg/L; negative results for ANA, ANCA, anti‐dsDNA antibodies, AMA and rheumatoid factor (RF) 0; CA 125 of 44 u/mL; absence of IgM anti‐EBV and anti‐CMV; and negative Widal and Wright test and of oval, vivax, falciparum plasmodium serodiagnosis. Whole‐body computed tomography (CT) showed modest liver and spleen volume increase and para‐aortic and common iliac artery lymphadenopathy (max 2 cm), while sternal bone marrow aspiration showed reactive hyperplasia. Esophagogastroduodenoscopy (EGDS) revealed an enlarged ampulla of Vater with mild plasmacytic infiltrate and rare lamina propria eosinophilic granulocytic infiltrate in histologic examination. The empirical antibiotic therapy with amoxicillin–clavulanate, ceftriaxone, levofloxacin, teicoplanin, and gentamicin was ineffective. Her medical history includes surgical treatment of left femoral hernia when she was 10 years.

**Figure 1:**
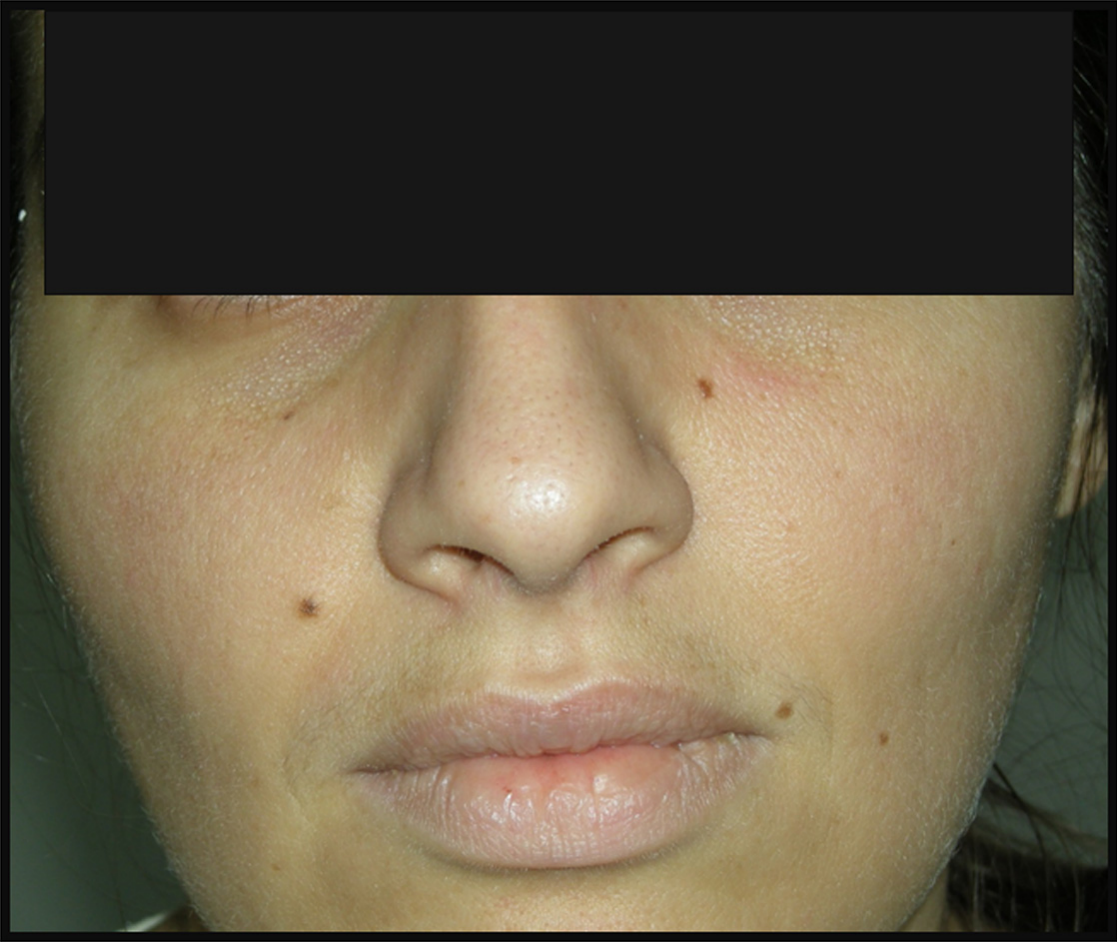
Evanescent, macular, erythematous (salmon‐pink) rash associated with fever episode.

On admission, blood pressure was 110/70 mmHg, heart rate 80 beats per minute, respiratory rate 16 breaths per minute, body temperature 38.5°C, and oxygen saturation 92%, while the patient was breathing ambient air. She was pale with nonpruritic, evanescent, macular, erythematous (salmon‐pink) rash (Fig. 1) alert and oriented, pharyngeal erythema. Cardiac examination revealed 2/6 ejection murmur on aortic area. Abdomen was treatable with the lower liver and spleen borders of the 2 cm below the costal margin. Severe pelvic girdle pain with stiffness, functional limitation, and myalgia was present as well – the overall level of disease activity on a visual analog scale (VAS) was 3.

The laboratory data were as follows: total leukocyte count, 19,000/mm^3^ (4000–10,000 mm^3^); differential leukocyte count: neutrophils, 88% (40–80), and lymphocytes, 14% (20–40); hemoglobin, 6 g/dL (women's normal range: 12–16); platelets, 740,000/mm^3^ (150,000–400,000/mm^3^); erythrocyte sedimentation rate (ESR), 106 mm/h (RR 0–43 mm/h); CRP, 214 mg/L (RR <5 mg/L); ferritin, 342 ng/mL (RR 8–252 ng/mL); serum iron, 2 mg/dL (RR 50–170 mg/dL); aspartate aminotransferase (AST), 43 U/L (RR 10–31 IU/L); alanine aminotransferase (ALT), 79 IU/L (RR 10–34 IU/L); alkaline phosphatase (ALP), 205 IU/L (RR 30–120 IU/L); g‐glutamyl transferase (GGT), 277 IU/L (RR 3–38 IU/L); antinuclear antibodies (ANA), 1:80 (absence); anti‐dsDNA antibodies and extractable nuclear antigen (ENA) antibodies negative; rheumatoid factor (RF), 12.5 IU/L (RR 0–25 IU/L); anticitrullinated protein antibodies (ACPAs), 0 U/mL (RR 0–6.9 U/mL); Quantiferon TB GOLD TEST: indeterminate (negative); and blood and urine cultures: negative. Additional laboratory markers are shown in Table 1.

**Table 1:** Laboratory data at admission, at discharge and at follow‐up

General laboratory tests	Adult reference range	Admission results	Post‐therapy results
Total leukocyte count (mm^3^)	4000–10,000	19,000	13.03
Differential leukocyte count (%)			
Neutrophils	40–80	88	66
Lymphocytes	20–40	14	26
Erythrocyte count	4,300,000–5,500,000	2,580,000	–
Hemoglobin (g/dL)	12–16 (women)	6	–
Hematocrit (%)	36–46 (women)	22.1	–
Platelet (mm^3^)	150,000–400,000	740,000	311,000
ESR (mm/h)	0–43	106	2
CRP (mg/L)	0–5	214	1
Procalcitonin (mg/L)	0–0.5	0.49	–
INR	0.8–1.2	1.17	–
Ferritin (ng/mL)	8–252	342	44
Serum iron (mg/dL)	50–170	2	20
AST (IU/L)	10–31	43	8
ALT (IU/L)	10–34	79	18
ALP (IU/L)	30–120	205	30
GGT (IU/L)	3–38	277	20
LDH (IU/L)	0–248	108	–
Serum albumin (g/dL)	3.5–5.2	2.8	3.8
	Reference Range	Result	
Markers of infection disease			
VDRL	Negative	Negative	–
HBsAg	Negative	Negative	–
Anti‐HCV antibodies	Negative	Negative	–
Anti‐HIV antibodies	Negative	Negative	–
Anti‐Coxsackie virus B1–B6	Negative	Negative	–
Blood cultures	Negative	Negative	–
Urine culture	Negative	Negative	–
Quantiferon TB GOLD TEST	Negative	Indeterminate	–
Markers of autoimmune disease			
ANA	Negative	1:80	–
Anti‐dsDNA antibodies	Negative	Negative	–
ENA	Negative	Negative	–
Complement C3 (g/L)	0.9–1.8	1.77	–
Complement C4 (g/L)	0.1–0.4	0.3	–
RF (IU/L)	0–25	12.5	–
ACPAs (U/mL)	0–6.9	0	–
Angiotensin‐converting enzyme (IU/L)	8–52	22	–
Urinary calcium (mg/24 h)	<250 (women)	33	–
*β*_2_ microglobulin (mg/dL)	0.1–0.25	0.1	–
Tumor Markers			
AFP (ng/mL)	0–8.1	0.1	–
CEA (ng/mL)	0–2.5	0.1	–
CA 125 (IU/mL)	0–35	38	–
CA 15.3 (IU/mL)	<32.4	12	–
CA 19.9 (IU/mL)	<37	2.9	–
NSE (*μ*g/L)	0–18.3	5.7	–
Fecal occult blood (ng/mL)	0–100	1	–

**Figure 2:**
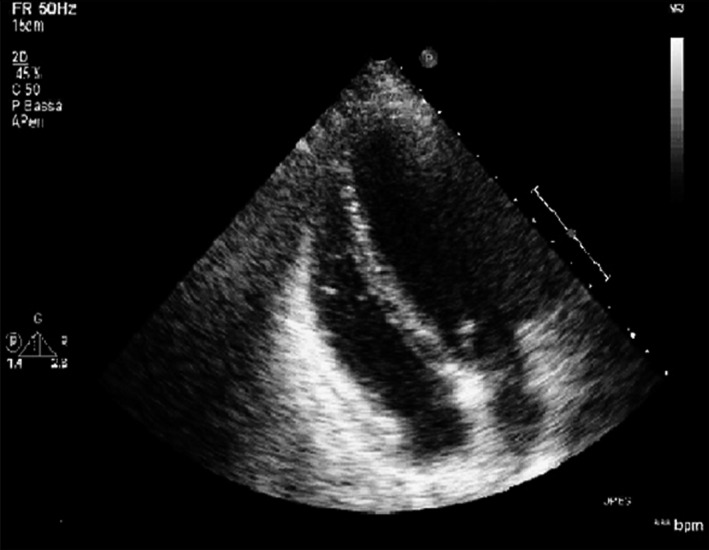
Transthoracic (TT) echocardiogram showing diffuse transmural hyperechoic myocardium and small circumferential pericardial detachment with hyperechoic aspect of the pericardium more evident on right sections.

ECG highlighted nonspecific anterolateral repolarization disorders. Echocardiogram (Fig. 2) showed diffuse transmural hyperechoic aspect of myocardium and small circumferential pericardial detachment with hyperechoic aspect of the pericardium, more evident on right sections. Head, chest, abdomen and pelvis contrast‐enhanced CT was similar to the previous.

The admission diagnosis to our department was fever of unknown origin 1; nevertheless, clinical presentation, patient's age, and positivity of Yamaguchi's criteria led to a working diagnosis of adult‐onset Still's disease 2, 3, 4. Specifically, she fulfilled the four major Yamaguchi's criteria – fever >39°C, ≥1 week; arthralgias >2 weeks; nonpruritic, evanescent, macular, erythematous (salmon‐pink) rash; WBC > 10,000 (>80% granulocytes); and five of the five minor ones – sore throat, lymphadenomegaly, hepatosplenomegaly, abnormal liver functional tests, and negative rheumatoid factor/antinuclear antibodies (<1:100) 5.

Adult‐onset Still's disease is an exclusion diagnosis among infectious, neoplastic, and rheumatic diseases 5; the last two were excluded by CT, EGDS, tumor markers, and negative autoimmune pattern.

**Figure 3:**
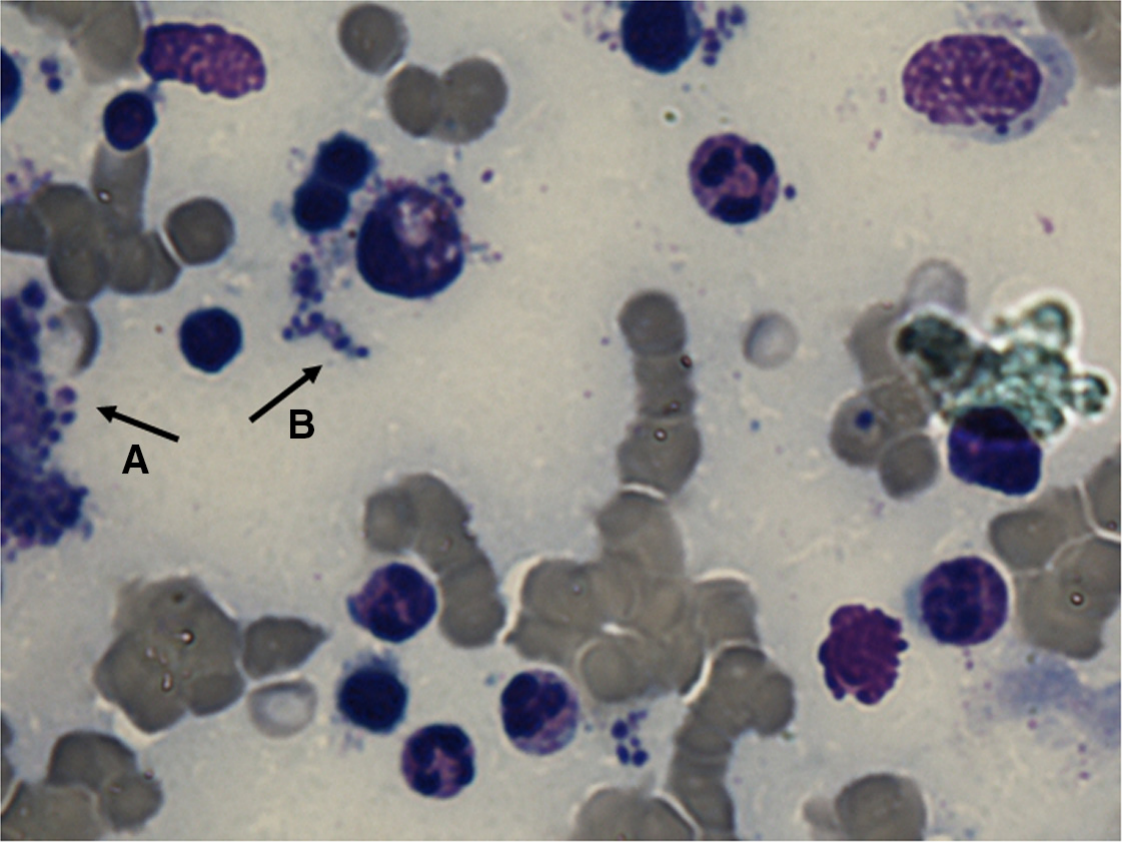
Bone marrow aspiration (Giemsa staining, x1000): Numerous amastigotes of *Leishmania spp.,* probably *infantum,* are mainly grouped in isles (A), also very massive in chain (B). Arrows indicate amastigotes. Original magnification (Giemsa staining 1000x).

During the workup of FUO with severe anemia, assuming AOSD, in a patient living in a leishmaniasis endemic area, we performed revaluation of sternal bone marrow aspiration searching and detecting amastigotes (Figure 3). Therefore, an anti‐Leishmania antibody titer (indirect immunofluorescence assay method) was requested showing 1:160, while polymerase chain reaction (PCR) with specific primers confirmed the presence of *Leishmania spp*. 6.

*Leishmania infantum*, responsible for visceral disease in the Mediterranean region with a reservoir in the domestic dog, could be the causative agent of patient's disease. In fact, patient lived near a cemetery frequented by stray dogs. Therefore, the patient was treated with intravenous liposomal amphotericin B (3 mg/kg IV once daily), replaced by pentamidine isethionate (4 mg/kg IV every other day), dexamethasone (6 mg IV), and promethazine prophylaxis for angioedema and wheezing appearance. A promptly clinical improvement followed this latter treatment.

The following gradual corticosteroid anaphylactic prophylaxis reduction led to a relapse of remittent fever (peak 40°C), sweating, arthralgia, myalgia, evanescent, macular, erythematous (salmon‐pink) rash, dry cough, and persistent elevation of inflammatory markers. This symptom relapse excludes reactive arthritis 7 and led to an AOSD diagnosis, according to Yamaguchi's criteria 5. Therefore, prednisone (1 mg/kg PO) was associated, following the diagnosis of secondary AOSD. The patient was treated with twenty pentamidine isethionate doses and a month of steroid therapy with slow taper off. Peripheral blood anti‐*Leishmania* antibodies and PCR and sternal bone marrow aspiration resulted negative at the end of specific therapy. Her overall condition and laboratory results significantly improved, ESR and CRP normalized (Table 1), and myocardial hyperechogenicity and pericardial effusion reduced.

The final clinical and pathological diagnosis was as follows: adult‐onset Still's disease caused by visceral leishmaniasis.

At one‐year follow‐up, the patient was in healthy condition.

## Discussion

Only two *Leishmania* species are endemic in European countries. *L. infantum* has a reservoir in the dogs and is responsible for zoonotic cutaneous and systemic disease within the Mediterranean region and for visceral disease in the Mediterranean basin and South America. *L. tropica* spreads mainly in Greece causing sporadic cases of anthroponotic cutaneous disease 8.

Visceral leishmaniasis has an annual incidence of 400,000 cases, with about 20,000–40,000 associated deaths and high degree of underreporting cases 9.

Leishmaniases are divided into three medical conditions that involve cutaneous, mucocutaneous, or visceral pathology.

Visceral leishmaniasis has a variable asymptomatic incubation period with intermittent fever, malaise, and shivering for early symptoms and anemia, leucopenia, and hepatosplenomegaly for overt disease 9.

Liposomal amphotericin B has the best safety profile and has been approved by the Food and Drug Administration (FDA) for the treatment of VL 10. Therefore, it is the most used drug in leishmaniasis species‐directed therapy in travelers 11.

Adult‐onset Still's disease is a rare systemic inflammatory disorder of unknown etiology and pathogenesis, characterized by quotidian or double‐quotidian spiking fevers with evanescent rash, arthritis, and multiorgan involvement. The disease is responsible for a significant proportion of FUO cases and affects mainly younger people between 16 and 35 years of age (three quarters of the patients) 12, 13, 14.

A very accredited hypothesis postulates a reactive component of AOSD with various infectious agents as disease triggers in a genetically predisposed host 1, 4, 5, 6, 7. Several factors such as genetics and bacterial and viral infections and environmental factors could have a causative role 12, 13, 14, 15, 16, 17. Viruses such as rubella, mumps, echovirus 7, cytomegalovirus, Epstein–Barr virus, parainfluenza, Coxsackie B4, adenovirus, influenza A, human herpesvirus 6, parvovirus B19, and hepatitis B and C have all been implicated in case reports and small series 12, 13, 14, 15, 16. Other studies have proposed microbial triggers including Mycoplasma pneumoniae, Chlamydia pneumoniae, Yersinia enterocolitica 3 and 9, Brucella abortus, and Borrelia burgdorferi as AOSD pathogenesis 12, 13, 14, 15, 16, 17. The hypothesis that infectious agents may trigger AOSD suggests a similarity with reactive arthritis 12.

The AOSD diagnosis according to Yamaguchi's criteria 5 foresees the presence of five criteria (at least two major) with sensitivity of 96.2% and specificity of 92.1% 12, 13, 14, 15, 16, 17, 18. The major criteria include arthralgia or arthritis ≥2 weeks with swelling or limited range of motion; warmth, pain, stiffness in one or more joints, in the absence of other reason of arthralgia; persistent, remittent fever ≥39°C ≥ 1 week; typical rash (evanescent, macular, erythematous salmon‐pink rash usually associated with fever); and elevated leukocyte count (>10,000/mm^3^ with >80% granulocytes). Minor criteria comprehend sore throat; lymphadenopathy and/or spleen involvement (recent significant lymph node swelling and splenomegaly confirmed on palpitation or by echography); elevated liver function tests (transaminases and/or lactate dehydrogenase) not attributable to drug, toxicity or allergy; and negative tests for antinuclear antibodies and RF. Exclusion criteria comprehend infections, malignancies, and other rheumatic diseases.

Differential diagnosis of AOSD includes infections (viral infections – rubella, Epstein–Barr virus, cytomegalovirus, HIV, hepatitis B and C, Coxsackie, and parvovirus, infective endocarditis, Lyme disease, and tuberculosis); granulomatous diseases: sarcoidosis, Crohn's disease, and idiopathic granulomatous hepatitis; malignancy: leukemias and lymphomas; and connective tissue diseases: systemic lupus erythematosus (SLE), mixed connective tissue disease (MCTD), polyarteritis nodosa (PAN), Wegener's granulomatosis, and Takayasu's arteritis 19.

The clinical course can develop into three distinct patterns (self‐limited or monocyclic, intermittent or polycyclic systemic, chronic articular) with significant prognostic implications; each of them affects about one‐third of AOSD patients 12.

Elevated ferritin levels are a nonspecific but common feature for AOSD diagnosis 20. In this case report, only moderate ferritin increase was due to concomitant anemia with very low hemoglobin levels and severe iron deficiency. Furthermore, normal serum ferritin levels should not be used to rule out the diagnosis, although the glycosylated fraction of ferritin is a more specific marker of AOSD 12, 13, 14, 15, 16, 17, 18, 19, 20.

The systemic corticosteroid administration is mainly restricted to the management of extra‐articular manifestations of systemic arthritis 4, 18, 19 (high fever, severe anemia, pericarditis), with pericarditis present in 23% of cases 12, 13.

In conclusion, leishmaniasis is a systemic infectious disease with important public health implications concerning people living or visiting endemic areas such as Sicily or Italy.

Therefore, clinicians should be considered VL in the differential diagnosis of patients with fever, anemia, and hepatosplenomegaly.

The inadequate response to standard anti‐Leishmania therapy alone (pentamidine) and the clinical improvement secondary to corticosteroid addition confirm the hypothesis that AOSD was due to VL.

Single‐episode AOSD presentation is generally due to or triggered by infectious agents.

Diagnosis of AOSD requires exclusion of infectious; however, viruses and microbial have been implicated as triggers in AOSD pathogenesis.

To the author's knowledge, no previous AOSD cases secondary to *Leishmania* infection have been reported in the literature.

## Authorship

SS, SC, SA, MC, and GD: wrote and revised the manuscript. SS, SC, EV, and EC: performed the analysis of case data; all authors: contributed to data analysis, drafting, and clinical revision of the manuscript and agreed to be responsible for any aspect of the manuscript.

## Conflict of Interest

The authors declare that there is no conflict of interests regarding the publication of this paper.
